# Summer Intestinal Diseases of Children

**Published:** 1894-09

**Authors:** 


BUFFALO MEDICAL AND SURGICAL JOURNAL

A MONTHLY REVIEW OF MEDICINE AND SURGERY.

*EDITORS:*

THOMAS LOTHROP, M. D. - - WM. WARREN POTTER, M. D.

All communications, whether of a literary or business nature, should be addressed to the managing editor: 284 FRANKLIN STREET, BUFFALO, N. Y.

VOL. XXXIV. SEPTEMBER, 1894. NO. 2.

### SUMMER INTESTINAL DISEASES OF CHILDREN.

As THE Summer season draws near unto its close, with its cool nights and hot days, we are confronted with the annual visitation of diarrhea, dysentery and cholera infantum, that children under two years are especially liable to be seized with. At this time the medical journals are publishing the annual output of literature, good, bad and indifferent, on this subject. We speak thus of the quality of the publications because while much of it is excellent, yet a certain proportion is written by young or inexperienced physicians, whose chief aim in writing appears to be to advertise some special preparation of food or medicine that they have been employed to write up.

The fact is that, in the care of infants, the greatest stress is to be laid upon the prevention of the Summer enteric diseases. Unless the nurse or mother be skilled in all the methods of prevention, the doctor can be of little avail in his attempt to invoke the laws of cure. In other words, he must begin by instructing in and enforcing the most ultra principles of cleanliness. In bottle-fed children, which constitute a large majority of those suffering from these diseases, it is next to useless to give medicine unless the way therefor is prepared by establishing the most arbitrary lines of cleanliness. This means not only personal cleanliness as applied to the infant and its surroundings, including bed, clothing, napkins and everything employed in its toilet, but its feeding apparatus should be subjected to the most rigid scrutiny and all the known laws of aseptic cleanliness be applied to prevent infection. A single point overlooked will often cause fermentation, and fermentation is only a step short of infection. Colonies of bacteria are rapidly produced in an intestinal tract poisoned by ferments that afford habitats for these microörganisms and a nidus for the generation of toxines.

Patent feeding bottles with long tubes are an abomination, and inventors thereof deserve the severest castigation. A four or six ounce bottle, with a rubber nipple that can be turned inside out, is the only apparatus necessary, and in its very simplicity is constituted its great excellence. These can easily be kept clean by employing a weak solution of bicarbonate of sodium and further cleansing them in a boracic acid solution with a proper brush, just before filling for use. Duplicate or triplicate bottles and nipples should be in constant employ, so that a fresh set may be always in readiness.

But, in spite of the utmost care by the most scrupulously neat attendant, intestinal diseases will appear, and in these cases the cause must be looked for in the food itself, either as to quality or quantity, or in the further fact that slight fermentation has begun before ingestion. It is often very difficult to detect this in milk or to prevent it unless it is sterilized. When traveling with infants, under an ever-changing environment, it is almost impossible to obtain ideal conditions. Boiled milk or artificial food must be employed during the period of touring.

In this brief statement it has not been our aim to discuss details, but rather to refer to a few well-established principles, our object being to accentuate the importance of prevention and not to enter into a lengthy dissertation on treatment. The latter is elaborately set forth in works on pediatrics, and most physicians are familiar with the principles that apply in the therapeutic management of these cases.

As a final word, we may remark that it is of the greatest importance that infants afflicted with these maladies shall be properly clothed. Light, soft flannel should be the chief texture worn, and great care should be exercised in keeping the extremities warm and in combating the tendency to overload the infant with too much bedclothing. First, last and always, they should have plenty of fresh air; hence, carefully ventilated apartments by night, and perambulating in the open air by day, should be a never violated rule.

